# Supplementary material for: Contact chains of cattle farms in Great Britain
Source: R Soc Open Sci. 2019 Feb 27;6(2):180719. doi: 10.1098/rsos.180719 (PMC6408381; doi:10.1098/rsos.180719)
Supplement: Cattle contact chains supplementary material [file rsos180719supp1.docx]

**Supplementary materials**

**Contact chains of cattle farms in Great Britain**

Helen R. Fielding^a^, Trevelyan J. McKinley^b^, Matthew J. Silk^a^, Richard J. Delahay^c^, and Robbie A. McDonald^a^*

^a^ Environment and Sustainability Institute, University of Exeter, Penryn Campus, Penryn TR10 9FE, Cornwall, United Kingdom.

^b^ College of Engineering, Mathematics and Physical Sciences, University of Exeter, Penryn Campus, Penryn TR10 9FE, Cornwall, United Kingdom.

^c^ Animal and Plant Health Agency, Woodchester Park, Nympsfield, Stonehouse GL10 3UJ, Gloucestershire, United Kingdom

*Corresponding author. *Email:* [r.mcdonald@exeter.ac.uk](mailto:r.mcdonald@exeter.ac.uk)

**Supplementary methods**

**Network analysis**

We generated random networks using the Erdös-Renyi model [1] to create 10,000 random networks per year (2001–2015). Edge weights from the observed network were randomly allocated to edges in the random networks for each respective year. We calculated the stability of the network position of individual farms across years using the methods of Wilson et al. [2]. For this analysis, we used nodes present in all years from 2001–2015, and within each year farms were ranked by the value of each network measure; in-degree, out-degree, in-strength, out-strength, and betweenness. We compared the standard deviation (SD) of ranks to the SD of ranks following node-based permutations (the swapping of attribute values between nodes of the network) of each yearly network, for each network measure. If the observed SD was lower than, and lay outside of the 95% confidence interval of, the SDs calculated from randomised networks, it was deemed to be repeatable through time. We additionally calculated what aspects of network position (in-degree, out-degree, in-strength, out-strength, and betweenness) correlated with mean herd size using Spearman’s rank correlation coefficients. To account for the non-independence of network data, we calculated equivalent Spearman’s rank correlations following node-based permutations for each year.

**Contact chains**

Using the same method employed to assess the stability of network measures on individual farms, we ranked ICC and OCC over all study years. In addition, using the same method used for correlations of mean herd size and network measures, we calculated the correlation of ICC with OCC, mean herd size with ICC and OCC, and node measures (in-degree, out-degree, in-strength, out-strength) with their corresponding ICC or OCC. Spearman’s rho (r_s_) and p-values were obtained for each individual study year and r_s_ is reported as a mean of all study years, with standard deviation.

**Supplementary results**

**Temporal variation in contact chains**

To investigate the variation in the contact chains of individual farms over time we performed additional analysis to assess our different methods. We compared the mean, median and maximum of the combined 24 monthly-spaced chains and the three combined annually-spaced chains (Figure S2) for years 2012 to 2014 using Spearman’s rank analysis. The majority of farms showed little variation in the number of farms in their chains between different starting months of the 24 monthly-spaced chains. Farms with around 10,000 farms in their chain seem to exhibit a much larger degree of variation than those with chain lengths both below and above them (Figure S7). We were concerned that seasonal fluctuations in movements would cause variation in the number of farms in a contact chain depending on the starting month of the chain. However, no consistent pattern was apparent by visual examination of monthly differences in the number of farms in contact chains from 2012-2014. This suggests that the variation seen in farms with contact chains of around 10,000 is due to individual farm trading behaviour rather than population-level changes in movements. Overall, there was a strong correlation between the summary values (mean, median and maximum) of the 24 monthly-spaced chains and the 3 annually-spaced chains (r_s_ (2.5 and 97.5 confidence intervals): mean = 0.932 (0.931–0.0.933), 0.909 (0.908–0.910), median = 0.845 (0.843–0.847), 0.846 (0.843–0.848), maximum = 0.923 (0.922–0.924), 0.874 (0.872–0.875) for ICC and OCC respectively; p < 0.001, n = 76,031).

**References**

1. Erdös P, Rényi a (1959) On random graphs. Publ Math 6:290–297 . doi: 10.2307/1999405

2. Wilson ADM, Krause S, Dingemanse NJ, Krause J (2013) Network position: A key component in the characterization of social personality types. Behav Ecol Sociobiol 67:163–173 . doi: 10.1007/s00265-012-1428-y

**Supplementary figures**
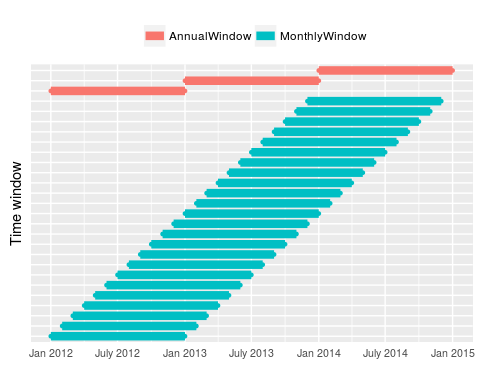


Figure S1. Schematic showing the two different time windows over which contact chains have been analysed and compared. Teal coloured bars span the 24 x 12-month periods from which movements are taken to create the sequential monthly annual contact chains. Red coloured bars span the 3 x 12-month periods within the selected period from which movements are taken to calculate annual contact chains.


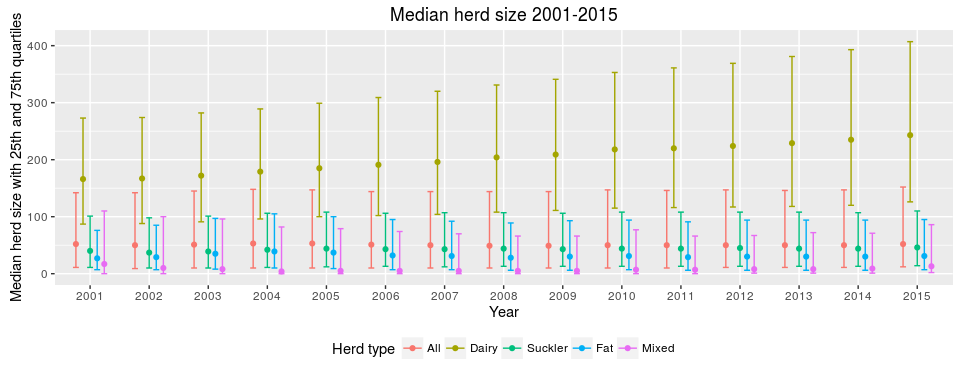


Figure S2. Summary measures for average herd sizes (mean number of cattle on premises over the year) for each year from 2001 to 2015 and for each herd type calculated from CTS data. Data are shown as boxplots with dots representing median values and whiskers representing interquartile ranges showing an increase in the size and variation of dairy herds and stability in the herd size of other herd types.


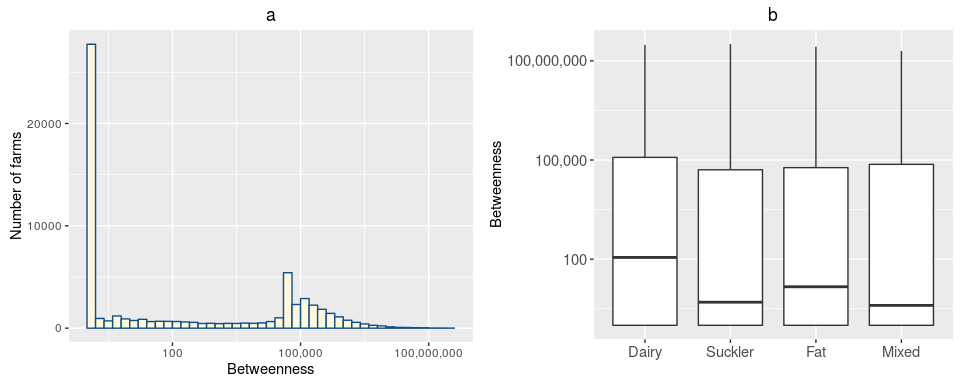


Figure S3: Individual node betweenness centrality calculated using inverted edge weights: a) Histogram with count log transformed (n+1) to show peaked distribution of betweenness and zero-inflated distribution, b) boxplot by herd type for the 2015 network of cattle movements showing little difference between herd types.


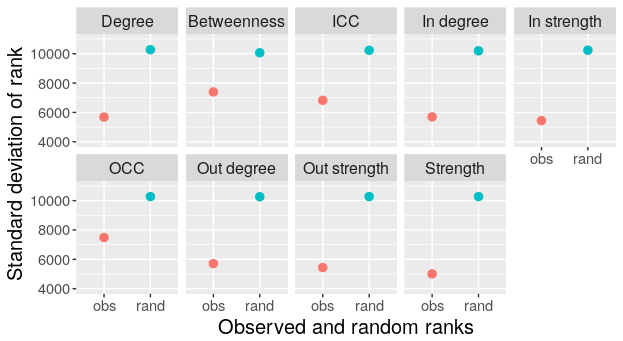


Figure S4: Observed standard deviation in farm ranks, when ranked by network measure over multiple years. Randomised standard deviation is from 1000 randomly generated Erdös-Renyi networks. In each case, observed standard deviation is less than random, indicating that farms were more consistent over time in their network measures than if the network were randomized.


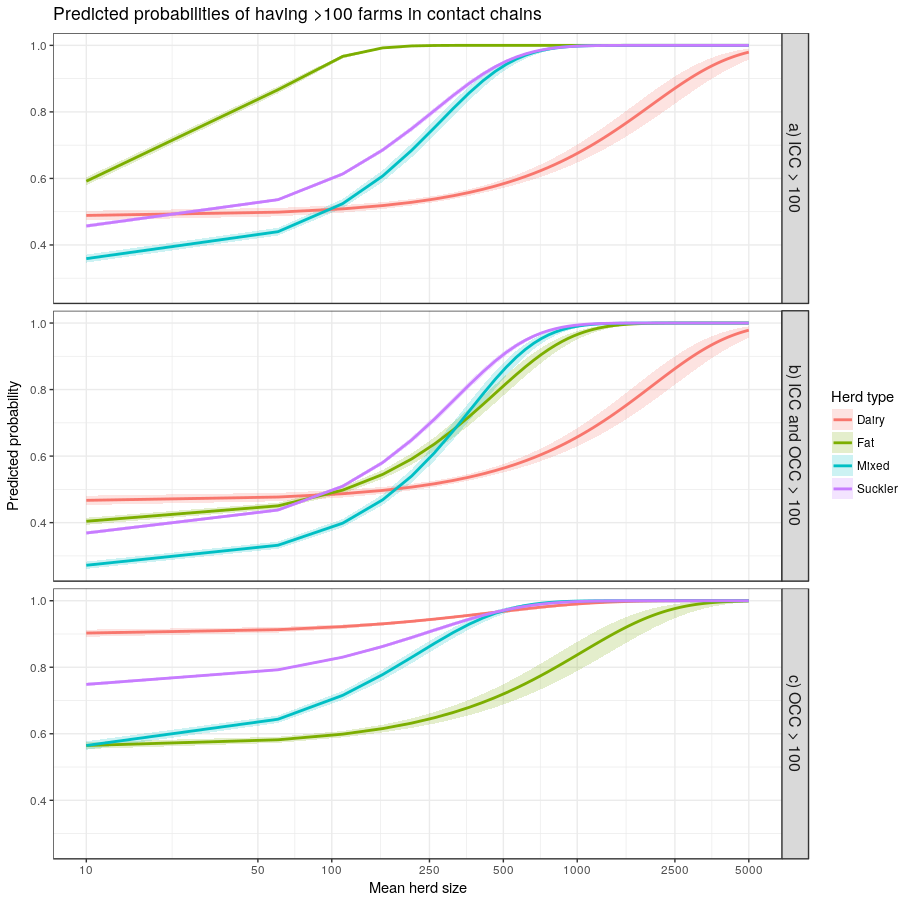
Figure S5: Predicted probabilities from logistic regressions using mean herd size and herd type as explanatory varia*bles with farms with over 100* farms in their a) ICC, b) both ICC and OCC and c) OCC as the response variable. Shaded areas represent the 95% confidence intervals. Uses the mean value of 24 sequential monthly-spaced contact chains from 2012-2014 as the value for contact chains.


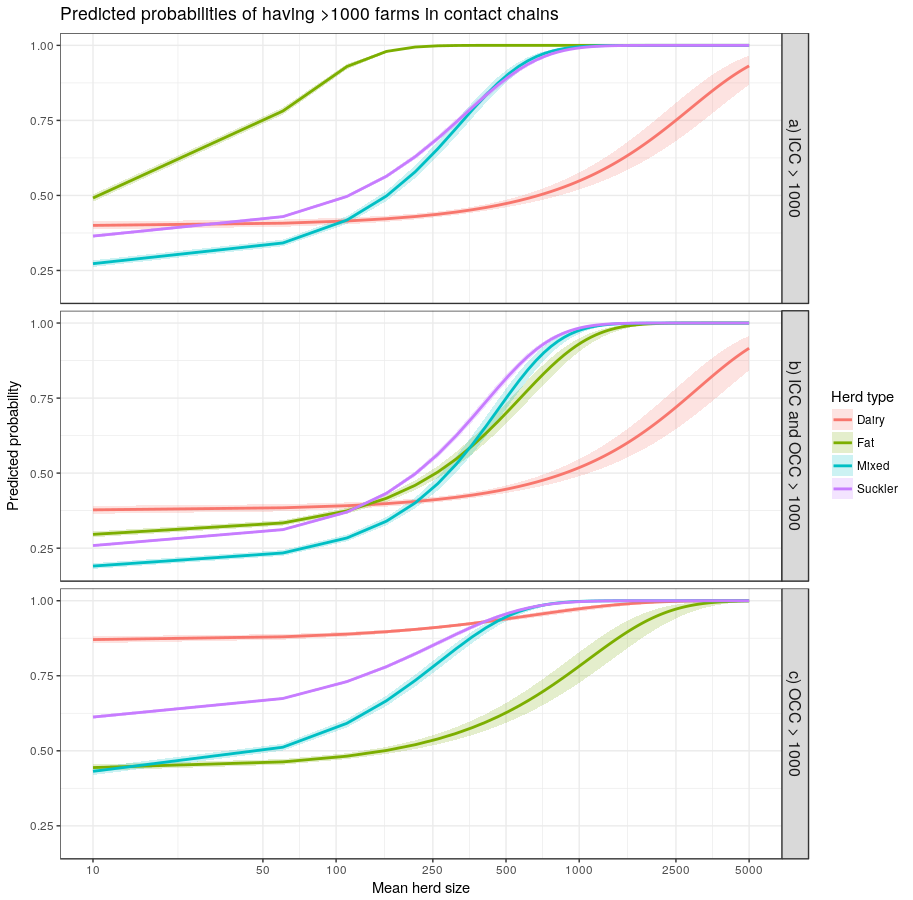
Figure S6: Predicted probabilities from logistic regressions using mean herd size and herd type as explanatory variables with farms with over 1000 farms in their a) ICC, b) both ICC and OCC and c) OCC as the response variable. Shaded areas represent the 95% confidence intervals. Uses the mean value of 24 sequential monthly-spaced contact chains from 2012-2014 as the value for contact chains.


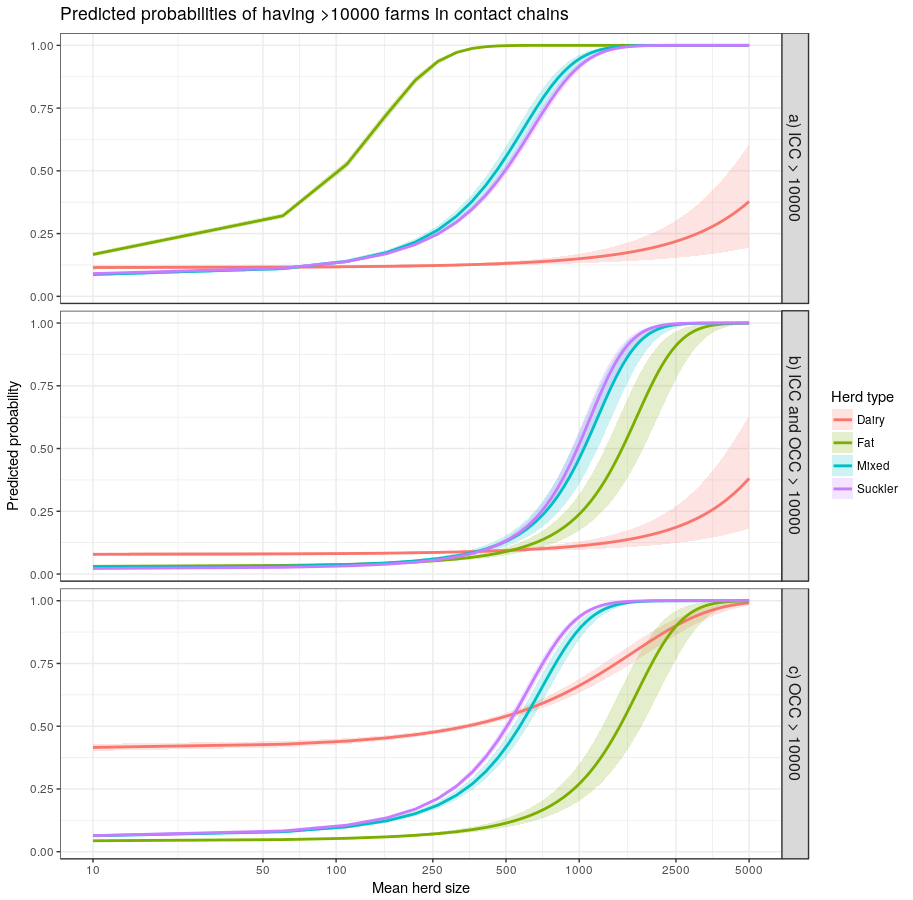


Figure S7: Predicted probabilities from logistic regressions using mean herd size and herd type as explanatory variables with farms with over 10,000 farms in their a) ICC, b) both ICC and OCC and c) OCC as the response variable. Shaded areas represent the 95% confidence intervals. Uses the mean value of 24 sequential monthly-spaced contact chains from 2012-2014 as the value for contact chains.


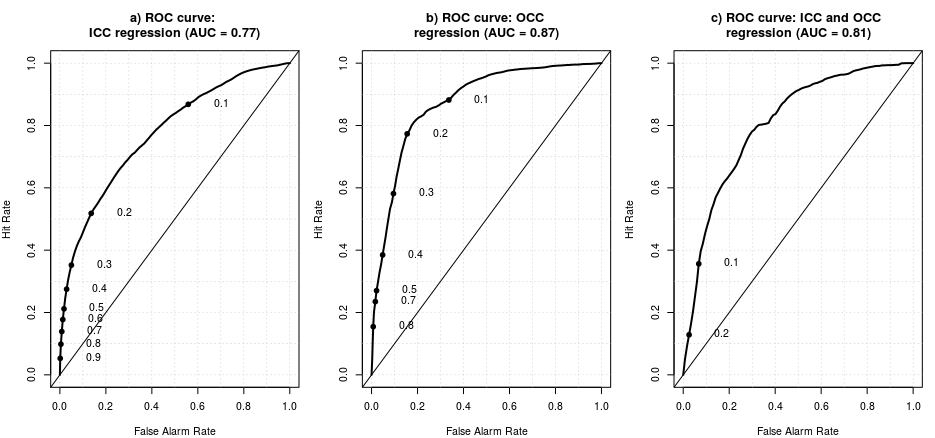


Figure S8: Receiver Operating Characteristic (ROC) curves for logistic regression using farms with over and under 10,000 farms in their a) ICC, b) OCC and c) ICC and OCC as response variables. The area under the curve (AUC) indicates discriminatory power of each model (0.70-0.80 = adequate, 0.80-0.90 = excellent): 0.77, 0.87 and 0.81 respectively.


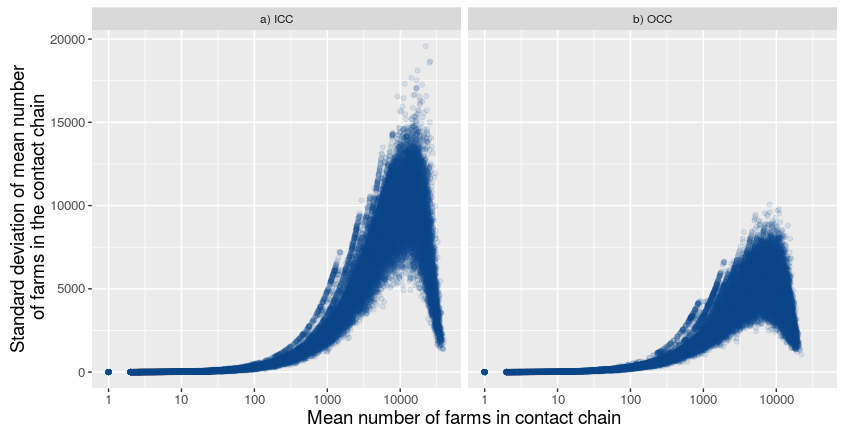


Figure S9: Relationship between standard deviation and the mean of 24 monthly-spaced annual a) ICCs and b) OCCs of all active farms in GB over the period 1st January 2012 to 31st December 2013. Showing that variation between contact chains of individual farms increases as the mean contact chain increases, but then decreases for farms at the extreme right tail of the distribution with over 10,000 farms in their chain.


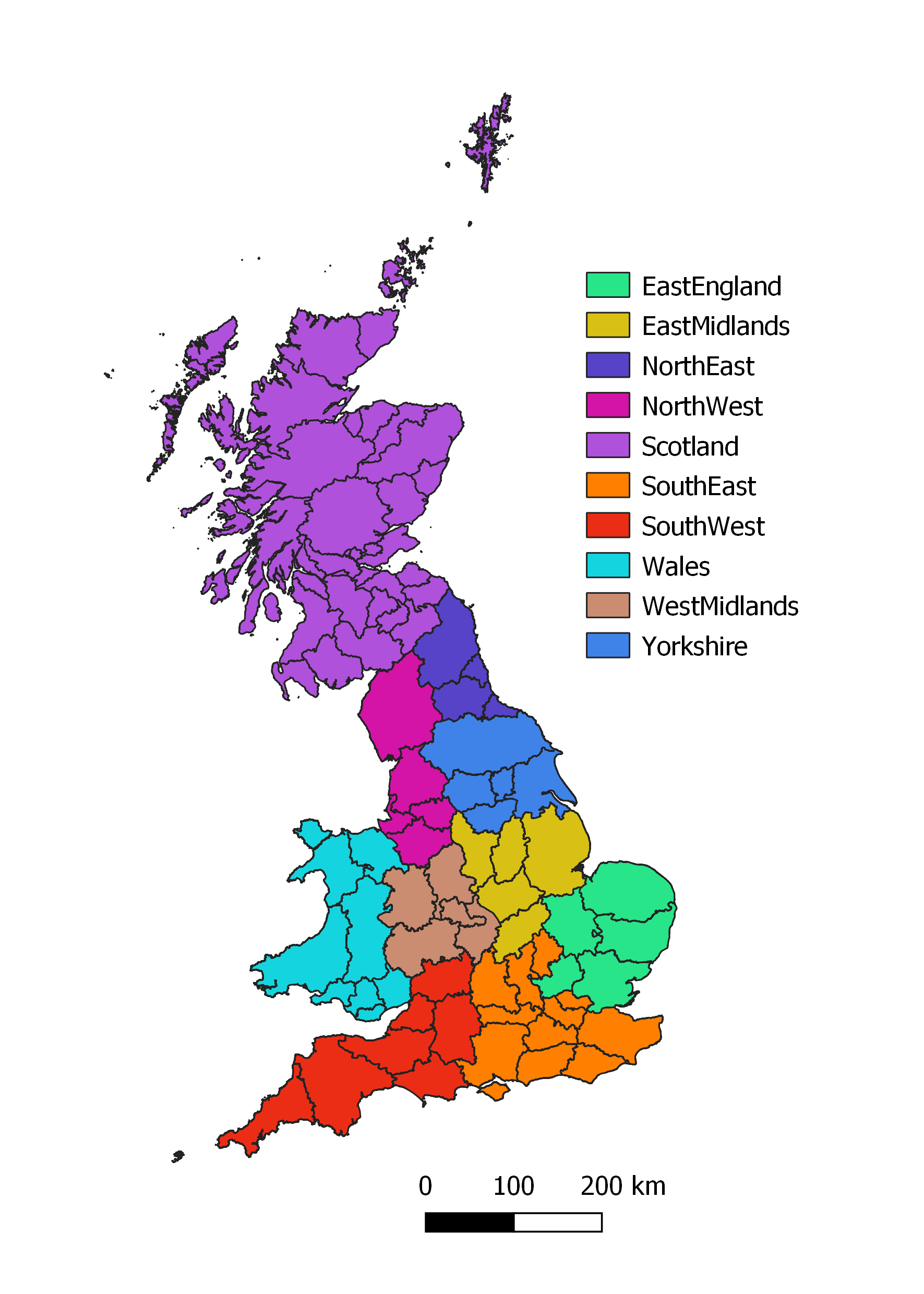


Figure S10: Map of Great Britain showing counties grouped into regions and countries used in our logistic regression analyses.

**Table S1**. Glossary of network analytical terms and accompanying igraph functions.

| Network measure | igraph function | Description |
| --- | --- | --- |
| *Shortest path* | - | The path between two nodes that traverses the least number of edges in the network. |
| *Betweenness centrality* | estimate_betweenness()* | A global measure of centrality indicating the number of times a node lies on the shortest-paths between all other nodes [1] in the network. Note* *In weighted networks, edge weights influence these shortest paths and betweenness values. For disease transmission, edges with high weight (more animals moved) represent an increased risk of pathogen transfer* [2]*. However, the algorithm used to calculate weighted betweenness by igraph treats high weight as a cost to edge traversal, therefore, we calculated betweenness with inverted weights (1/weight) to represent this higher risk of transmission.** |
| *Edge density* | edge_density() | Defined as the ratio between the number of edges in the network and all possible edges giving a value for density that is relative to the number of nodes. In general, a more dense network has more edges along which transmission can occur [3]. |
| *Degree assortativity* | assortativity_degree() | The extent to which nodes connect with those with a similar degree to them and gives an indication of the presence of hubs in the network, i.e. those nodes that connect to many more other nodes than the majority. Hubs facilitate rapid spread of infection to their connected nodes, temporarily increasing the speed of epidemic spread [4]. |
| *Reciprocity* | reciprocity() | Indicates the extent to which nodes reciprocally connect, by both buying and selling, to one another in a directed network. |
| *Clustering coefficient* | transitivity() | The *clustering co-efficient* detects network clustering and shows the tendency for nodes to be connected to other nodes that are themselves directly connected. |
| *Average path length* | average.path.length () | The average of all shortest paths in the connected network. It indicates in how many steps the GSCC can be traversed. |
| *Giant strongly-connected component* (GSCC) | strong <-components(g, mode='strong')  gscc <- max(strong$csize) | The largest group of connected farms from which any node may reach any other node via directed links [5], and has been used to estimate the potential extent of an epidemic [6]. |
| *Small-world networks* | - | Low density networks with high clustering exhibit *small-world* type properties, which can increase disease transmission but may reduce the spatial extent of the epidemic [7]. |
| *Scale-free networks* | - | Small-world type networks with a power-law [8] degree distribution are considered to be *scale-free*. Specifically in scale-free networks disease spread can occur regardless of epidemic thresholds, which often govern the spread of disease in other network structures [9]. This can facilitate the transmission of infections with low reproductive rates, and therefore makes a population more susceptible to a greater range of pathogens. |

1. Newman MEJ. 2015 *Networks: An Introduction*. 2nd edn. Oxford University Press.

2. Natale F, Savini L, Giovannini A, Calistri P, Candeloro L, Fiore G. 2011 Evaluation of risk and vulnerability using a Disease Flow Centrality measure in dynamic cattle trade networks. *Prev. Vet. Med.* **98**, 111–118. (doi:10.1016/j.prevetmed.2010.11.013)

3. Shirley MDF, Rushton SP. 2005 The impacts of network topology on disease spread. *Ecol. Complex.* **2**, 287–299. (doi:10.1016/j.ecocom.2005.04.005)

4. Kiss IZ, Green DM, Kao RR. 2006 Infectious disease control using contact tracing in random and scale-free networks. *J. R. Soc. Interface* **3**, 55–62. (doi:10.1098/rsif.2005.0079)

5. Pastor-Satorras R, Castellano C, Van Mieghem P, Vespignani A. 2015 Epidemic processes in complex networks. *Rev. Mod. Phys.* **87**, 925–979. (doi:10.1103/RevModPhys.87.925)

6. Kiss IZ, Green DM, Kao RR. 2006 The network of sheep movements within Great Britain: Network properties and their implications for infectious disease spread. *J. R. Soc. Interface* **3**, 669–677. (doi:10.1098/rsif.2006.0129)

7. Christley RM, Pinchbeck GL, Bowers RG, Clancy D, French NP, Bennett R, Turner. 2005 Infection in Social Networks: Using Network Analysis to Identify High-Risk Individuals. *Am. J. Epidemiol.* **162**, 1024–1031. (doi:10.1093/aje/kwi308)

8. Clauset A, Rohilla Shalizi C, J Newman ME. 2009 Power-Law Distributions in Empirical Data. *SIAM Rev.* **51**, 661–703. (doi:10.1214/13-AOAS710)

9. Pastor-Satorras R, Vespignani A. 2001 Epidemic spreading in scale-free networks. *Phys. Rev. Lett.* **86**, 3200–3203. (doi:10.1103/PhysRevLett.86.3200)

**Table S2.** Observed network values for cattle movement networks in Great Britain 2001–2015. The p-values indicate the probability of the values calculated in the 10,000 Erdös-Renyi random networks being greater than the equivalent value calculated from the observed network. We applied a Bonferroni correction to α=0.05 to account for the fact that we tested the same network values over 15 years. Therefore, p values are considered statistically significant when p<0.003 or p>0.997.

Table S3: ROC values for logistic regression models calculated at different thresholds as a response variable for the number of farms in a contact chain.

| ROC value | Threshold number of farms in response variable | | |
| --- | --- | --- | --- |
| Chain | 100 | 1000 | 10000 |
| ICC | 0.71 | 0.71 | 0.77 |
| OCC | 0.75 | 0.76 | 0.87 |
| ICC and OCC | 0.68 | 0.69 | 0.81 |

Table S4: Power law exponents from the degree distribution in the observed networks from 2001–2015. Test statistics are from a likelihood ratio test performed to compare models that fitted the observed network to a power-law distribution with that from a log-normal distribution. In all years, apart from 2001 when trading patterns were grossly perturbed by movement restrictions, there was no evidence that either model fitted better.

| Year | Power-law exponent | R test statistic (log-likelihood ratio) | One-sided p-value |
| --- | --- | --- | --- |
| 2001 | 3.14 | 4.9940 | 2.96E-07 |
| 2002 | 3.00 | 0.0321 | 0.487 |
| 2003 | 2.91 | -0.7544 | 0.775 |
| 2004 | 2.89 | -0.5700 | 0.716 |
| 2005 | 2.82 | -0.2211 | 0.587 |
| 2006 | 2.71 | -0.7489 | 0.773 |
| 2007 | 2.76 | -0.1060 | 0.542 |
| 2008 | 2.77 | 0.0019 | 0.499 |
| 2009 | 2.77 | 0.4970 | 0.310 |
| 2010 | 2.78 | 0.4315 | 0.333 |
| 2011 | 2.75 | -0.2503 | 0.599 |
| 2012 | 2.81 | -0.0519 | 0.521 |
| 2013 | 2.78 | -0.2134 | 0.584 |
| 2014 | 2.80 | 0.0744 | 0.470 |
| 2015 | 2.83 | -0.2063 | 0.582 |
